# High Diversity, Prevalence, and Co-infection Rates of Tick-Borne Pathogens in Ticks and Wildlife Hosts in an Urban Area in Romania

**DOI:** 10.3389/fmicb.2021.645002

**Published:** 2021-03-09

**Authors:** Silvia-Diana Borşan, Angela Monica Ionică, Clémence Galon, Andra Toma-Naic, Cosmin Peştean, Attila D. Sándor, Sara Moutailler, Andrei Daniel Mihalca

**Affiliations:** ^1^Department of Parasitology and Parasitic Diseases, University of Agricultural Sciences and Veterinary Medicine of Cluj-Napoca, Cluj-Napoca, Romania; ^2^“Regele Mihai I al României” Life Sciences Institute, University of Agricultural Sciences and Veterinary Medicine of Cluj-Napoca, Cluj-Napoca, Romania; ^3^UMR BIPAR, Animal Health Laboratory, ANSES, INRAE, Ecole Nationale Vétérinaire d’Alfort, Paris-Est Sup, Maisons-Alfort, France; ^4^Department of Surgery, Anesthesiology and Intensive Therapy, University of Agricultural Sciences and Veterinary Medicine of Cluj-Napoca, Cluj-Napoca, Romania; ^5^Department of Parasitology and Zoology, University of Veterinary Medicine, Budapest, Hungary

**Keywords:** urban, hard ticks, wildlife hosts, tick-borne pathogens, co-infections

## Abstract

Despite the increasingly recognized eco-epidemiological importance of ticks as vectors for numerous zoonotic pathogens in urban areas, data regarding the pathogen diversity and co-infection rates in ticks and wildlife hosts in urban and peri-urban Romania are scanty. We aimed to establish the risk of human exposure to co-infected ticks in Cluj-Napoca, a major city in Romania. DNA was isolated from 151 questing ticks: *Ixodes ricinus* (*n* = 95), *Haemaphysalis punctata* (*n* = 53), *Dermacentor reticulatus* (*n* = 2), and *Dermacentor marginatus* (*n* = 1); 222 engorged ticks: *I. ricinus* (*n* = 164), *I. hexagonus* (*n* = 36), *H. punctata* (*n* = 16), *H. concinna* (*n* = 6), and 70 tissue samples collected from wildlife hosts during 2018 in five urban, and two peri-urban sites. Using a pre-designed Fluidigm real-time PCR dynamic array, all DNA samples were individually screened for the presence of 44 vector-borne pathogens. Subsequently, conventional PCRs were performed for a selection of samples to allow validation and sequencing. In total, 15 pathogens were identified to species and 6 to genus level. In questing ticks, single infections were more common than co-infections. Seven *Borrelia* spp. were detected in questing *I. ricinus*, and three in *H. punctata* ticks. An overall high prevalence 26.35% (95% CI: 19.46–34.22) and diversity of *Borrelia burgdorferi* sensu lato was seen in urban questing ticks. Other pathogens of the order Rickettsiales were present with variable prevalence. Co-infections occurred in 27.4% (95% CI: 18.72-37.48) of all infected questing ticks. In engorged ticks the overall *Bo. burgdorferi* sensu lato prevalence was 35.6% (95% CI: 29.29–42.27), with five species present. Pathogens of the order Rickettsiales were also frequently detected. We report for the first time in Romania the presence of *Rickettsia aeschlimannii* and *Rickettsia felis*. Overall, from the infected engorged ticks, 69.2% showcased co-infections. In *Ixodes* spp., dual co-infections, namely *Borrelia* spp. and *Anaplasma phagocytophilum*, and *Rickettsia helvetica* and *A. phagocytophilum* were the most prevalent. Given the outcome, we underline the need to establish proper tick-surveillance programs in cities and include co-infections in the management plan of tick-borne diseases in Romania.

## Introduction

Ticks are arthropods that can transmit pathogenic microorganisms including protozoa, bacteria, and viruses. In Europe, the majority of human and animal arthropod-borne diseases are vectored by ticks (Jongejan and Uilenberg, 2004; Colwell et al., 2011). Both humans and pets face a significantly higher risk of contracting tick-borne pathogens (TBPs) due to the emergence of ticks in urban areas (Rizzoli et al., 2014).

*Ixodes ricinus* is the predominant tick species reported in Europe (Rizzoli et al., 2014) and the most widespread questing tick species collected in Romania’s natural (Mihalca et al., 2012a) and urban habitats (Borşan et al., 2020). Moreover, it is also the most prevalent tick species reported to bite humans in Romania (Briciu et al., 2014; Andersson et al., 2018).

The habitat range of *I. ricinus* includes both natural and urban environments such as recreational areas, parks, and gardens, which can ensure the abiotic and biotic requirements for optimal development of the off-host stages (Rizzoli et al., 2014).

To date, a considerable number of studies describe the pathogens vectored by *I. ricinus* worldwide (Keesing et al., 2010; Rizzoli et al., 2014; Strnad et al., 2017). The TBPs which pose the greatest risk for the public health are the spirochetes of the *Borrelia burgdorferi* sensu lato complex, the causative agents of human Lyme borreliosis (LB), and the European tick-borne encephalitis virus, which can lead to tick-borne encephalitis (TBE) (Gritsun et al., 2003; Rizzoli et al., 2011). In Romania, various molecular approaches have been used to detect the prevalence of infection with the *Borrelia* spp. (including the relapsing fever spirochete *B. miyamotoi)* (Kalmár et al., 2016) in questing ticks (Kalmár et al., 2013; Raileanu et al., 2017), ticks collected from animal hosts (Gherman et al., 2012; Dumitrache et al., 2015; Kalmár et al., 2019), or humans (Andersson et al., 2014; Briciu et al., 2014; Andersson et al., 2018; Kalmár et al., 2020). During 2018 a total of 532 human Lyme disease cases were confirmed by serology in Romania (NCSCC, 2018), placing the country at the inferior position of the European incidence (Rizzoli et al., 2011). Other pathogens vectored by *I. ricinus* that are of rising importance for medical and veterinary health are bacteria of the order Rickettsiales. Despite the wide distribution of *Anaplasma phagocytophilum* across Romania (Matei et al., 2015), and of the presence of this pathogen in *I. ricinus* ticks collected from humans (Matei et al., 2017), no clinical human cases were reported so far in the country. Yet to be described from humans in Romania, nonetheless detected in questing (Kalmár et al., 2016) and engorged *I. ricinus* ticks collected from humans (Andersson et al., 2014; Kalmár et al., 2020), *Neoehrlichia mikurensis* is an emerging pathogen which can either lead to severe febrile illness in immunocompromised patients (Grankvist et al., 2014) or fever in clinically healthy humans (Li et al., 2012). The spotted fever group rickettsiae (SFG) cause rickettsioses in humans (Parola et al., 2013). In Romania, *R. conorii* (the Mediterranean Spotted Fever), *R. massiliae*, and *R. slovaca* and *R. raoultii* (SENLAT-scalp eschar and neck lymphadenopathy after a tick bite syndrome) were reported from human patients (Serban et al., 2009; Zaharia et al., 2016). While neither of these bacterial species are vectored by *I. ricinus*, several other *Rickettsia* spp. were also identified in ticks collected from wildlife hosts (Mărcuţan et al., 2016; Sándor et al., 2017) or the environment (Ioniţa et al., 2013). The role of *I. ricinus* is also suspected in the human transmission of *Bartonella* species such as *B. quintana* and *B. henselae* (Socolovschi et al., 2009; Vu Hai et al., 2014). This tick species is also a vector for zoonotic apicomplexans of the genus *Babesia*, such as *B. divergens* and *B. microti* (Gray et al., 2010). To date, *B. microti* and *B. venatorum* are reported in ticks collected from humans in Romania (Kalmár et al., 2020).

Due to the generalist feeding behavior of *I. ricinus*, co-infections with several micro-organisms are frequent in this tick (Reis et al., 2011). Multiple strains of bacteria, parasites, and viruses can be acquired by ticks either from a host with multiple infections, through feeding on subsequent hosts (along with the individual development), or through co-feeding mechanisms (Piesman and Happ, 2001). Transstadial, or in the case of some TBPs (i.e., *Borrelia* spp., *Rickettsia* spp., and TBE-complex virus), transovarial transmission in ticks can also contribute to the ecology of such pathogens (Sprong et al., 2009; Rizzoli et al., 2011; Karbowiak and Biernat, 2016). It is noteworthy that the transmission of pathogens from co-infected ticks is likely to alter the severity of clinical signs in humans or animals (Cutler et al., 2020), sometimes causing delays or errors in diagnosis as reported for concurrent babesiosis and Lyme disease (Grunwaldt et al., 1983; Golightly et al., 1989). Due to the potential implications of co-infections in urban ticks and the likelihood of co-transmission of TBPs it is vital to identify local enzootic cycles, especially in recreational areas.

Co-infection prevalence in ticks in European countries ranges from 3.2% to 45% (Reye et al., 2010; Reis et al., 2011; Lommano et al., 2012; Moutailler et al., 2016; Klitgaard et al., 2019; Nebbak et al., 2019; Kalmár et al., 2020). Little is known about the co-infection rates in questing ticks and wildlife hosts in urban and peri-urban Romania (Raileanu et al., 2017, 2018). Effective tick-based surveillance is essential for monitoring human and animal disease emergence. Therefore, by using a powerful broad-spectrum high-throughput approach, our study aimed to: 1. detect the TBPs in questing, engorged ticks, and in tissue samples from wildlife fauna collected in seven locations in Cluj-Napoca, Romania; 2. determine the co- infection rates; and 3. perform a comparative statistical analysis of infection rates and pathogen diversity in ticks from urban and peri-urban habitats.

## Materials and Methods

### Sampling Protocol

The characteristics of the urban and peri-urban locations assessed in this study, the method of collection of questing ticks, the sampling and trapping protocols for urban wildlife hosts and their associated tick fauna, followed by species-specific identification of all organisms as well as the research and ethical permits are described in detail elsewhere (Borşan et al., 2020). The five urban locations assessed during 2018 consisted of two parks: “Iuliu Haţieganu Park” and the campus of the “University of Agricultural Sciences and Veterinary Medicine of Cluj-Napoca (USAMV Campus); two gardens: “Alexandru Borza Botanical Garden” and a centrally located private garden; and “Mănăştur Cemetery”. The peri-urban sites were represented by “Hoia” and “Făget” forest. All locations (except the private garden) are open to the public year-round. The sampling activities were performed from March until November 2018 and included flagging (bimestrial) to collect questing ticks and collecting of wildlife using standardized methods (i.e., rodent trapping with “snap-traps”; bird sampling using ornithological mist nets, and hedgehog sampling by “torch-based” searches). All wildlife hosts were searched for ticks, blood samples were collected from birds and hedgehogs (if feasible), followed by release, while the trapped rodents underwent necropsy.

### Questing Ticks

From the 3383 total questing ticks collected during 2018, we randomly selected individual ticks for DNA isolation as follows: 10% (or 5 individual ticks if the numbers were too low to meet the 10% criteria) from each tick species and developmental stage (questing larvae were excluded) per location during each month of sampling. If the tick number (from the same species, stage, location, and month) was less than 5, all sampled ticks were included. A total of (*n* = 95) *Ixodes ricinus* (*n* = 77 ticks collected in urban, and *n* = 18 in peri-urban sites) and (*n* = 53) *Haemaphysalis punctata* ticks (*n* = 32 ticks collected in urban, and *n* = 21 in peri-urban sites) were selected. In addition, we included two individuals of *Dermacentor reticulatus* and one individual of *D. marginatus* accidentally collected in peri-urban locations during the flagging campaigns. Overall, 151 questing ticks were used for DNA isolation (Supplementary File 1).

### Urban Wildlife Hosts

All the engorged ticks collected from rodents and birds were individually tested, while in the case of ticks collected from hedgehogs (*Erinaceus roumanicus*), samples were selected using a similar algorithm as for questing ticks (including larvae). Therefore, we selected for DNA isolation 222 engorged ticks (*n* = 20 ticks from rodents; *n* = 22 ticks from birds; *n* = 180 ticks from hedgehogs) as follows: a total of 215 ticks consisting of *I. ricinus* (*n* = 157), *I. hexagonus* (*n* = 36), *H. punctata* (*n* = 16) and *H. concinna* (*n* = 6) collected from wildlife hosts found in urban sites, and 7 *I. ricinus* ticks found on hosts from peri-urban areas) (Table 1 and Supplementary File 1).

**TABLE 1:** Wildlife and associated tick species collected in Cluj-Napoca during 2018.

		**Tick species and developmental stage**					
**Location**	**Host species**	**Tick species**	**F**	**M**	**N**	**L**	**TOTAL**
Iuliu Haţieganu Park	*Erinaceus roumanicus* (*n* = 4)	*I. ricinus*	-	3	18	19	40
		*I. hexagonus*	10	2	–	1	13
		*H. punctata*	–	–	5	6	11
	*Apodemus agrarius* (*n* = 3)	*I. ricinus*	—	–	–	4	4
	*Garrulus glandarius* (*n* = 1)	*I. ricinus*	–	–	1	1	2
USAMV Campus	*Erinaceus roumanicus* (*n* = 4)	*I. ricinus*	9	5	16	17	47
		*I. hexagonus*	7	1	–	–	8
		*H. punctata*	–	–	3	–	3
	*Erithacus rubecula* (*n* = 1)	*I. ricinus*	–	–	1	1	2
	*Passer montanus* (*n* = 3)	*I. ricinus*	–	–	3	–	3
	*Phylloscopus collybita* (*n* = 1)	*I. ricinus*	–	–	1	–	1
	*Sturnus vulgaris* (*n* = 2)	*I. ricinus*	–	–	1	–	1
		*H. concinna*	–	–	6	–	6
	*Turdus merula* (*n* = 1)	*I. ricinus*	–	–	3	–	3
Alexandru Borza Botanical Garden	*Erinaceus roumanicus* (*n* = 3)	*I. ricinus*	6	7	15	15	43
		*I. hexagonus*	10	5	–	–	15
	*Apodemus flavicollis* (*n* = 1)	*I. ricinus*	–	–	–	5	5
	*Talpa europaea* (*n* = 1)	*I. ricinus*	–	–	–	4	4
	*Turdus merula* (*n* = 1)	*I. ricinus*	–	–	1	1	2
		*H. punctata*	–	–	–	2	2
Hoia forest	*Apodemus flavicollis* (*n* = 2)	*I. ricinus*	–	–	1	5	6
	*Sorex minutus* (*n* = 1)	*I. ricinus*	–	–	–	1	1

*F, females; M, males; N, nymphs; L, larvae.*

Following the visual inspection, all micromammals except hedgehogs underwent necropsy. During the necropsy, the heart, liver tissue, and two skin biopsies (one from the interscapular region and the second from the ear pavilion that were pooled together for DNA isolation) were collected from each animal. All the tissue samples were labeled accordingly and stored at −20°C. During the anesthesia -protocol described in Borşan et al. (2020), 0.5 ml of blood were collected from the jugular or saphenous vein of eight hedgehogs. Blood sampling was unsuccessful in the case of three hedgehogs. Regarding the birds (Borşan et al., 2020), a 150 μl blood sample was collected from the brachial vein using a microcapillary tube. All blood samples were stored in a 3.2% citrate tube at −20°C. Overall, DNA was individually isolated from the heart, liver, and skin biopsy tissues from 29 mammal hosts, 33 blood samples from birds, and eight blood samples from hedgehogs (Supplementary File 1).

### DNA Isolation

The genomic DNA isolation was performed individually for all tissues and tick samples using the Isolate II Genomic DNA Kit (Bioline, London, United Kingdom), according to the manufacturer’s instructions. Each tick was dried, cut into halves using a sterile scalpel blade, and crushed with a sterile pestle. For tissue samples, up to 25 mg of tissue was cut into small pieces and crushed with a sterile pestle, before the lysis. To ensure proper lysis, overnight digestion was performed for both ticks and tissue samples. The blood samples were processed using the same kit. A quantity of 200 μl of blood was used from the hedgehog samples, and 100 μl for birds. The DNA was stored at −20°C until further processing.

### Detection of Tick-Borne Pathogens

#### DNA Pre-amplification

The DNA pre-amplification steps were followed as described in (Michelet et al., 2014).

#### High-Throughput Real-Time PCR System

The BioMark real-time polymerase chain reaction (PCR) system (Fluidigm, San Francisco, CA, United States), was used for high-throughput microfluidic real-time PCR amplification using the 48.48 dynamic arrays. The chips dispensed 48 PCR assays and 48 samples into individual wells, after which on-chip microfluidics assemble PCR reactions in individual chambers before thermal cycling resulting in 2304 individual reactions (Michelet et al., 2014).

Subsequent pre-amplification, Real-Time PCR were performed using FAM- and black hole quencher (BHQ1)-labeled TaqMan probes with PerfeCTa qPCR ToughMix, Low ROX (QuantaBio, Beverly, MA, United States) following the protocol by Michelet et al. (2014). Thermal cycling conditions were as follows: 50°C for 2 min, 95°C for 10 min, 40 cycles of 2-step amplification at 95°C for 15 s, and 60°C for 1 min. Data were acquired on the BioMark Real-Time PCR system and processed using the Fluidigm Real-Time PCR Analysis software to obtain a cut-off (Ct) value (Michelet et al., 2014; Gondard et al., 2020).

The BioMark real-time PCR system (Fluidigm, San Francisco, CA, United States) was used for high-throughput microfluidic real-time PCR for the most common bacterial and parasitic TBP species known to circulate or recently emerging in Europe. The real-time PCR system developed for the screening of known and potential TBPs in Romanian ticks included 47 sets of primers and probes (Michelet et al., 2014; Sprong et al., 2019; Gondard et al., 2020). Among them, 37 primers were used for the detection of pathogens to species level (*n* = 30 bacterial and *n* = 7 apicomplexan) and 8 primers to genus level (*n* = 5 bacterial and *n* = 3 apicomplexan). Three sets of primers and probes were used for the molecular identification of two tick species found in Romania: *I. ricinus* and *D. reticulatus*. Lastly, a primer targeting a conserved region of the 16S rRNA gene in ticks, called “Tick spp.” was used as a control for the DNA extraction. To determine if factors present in the sample could inhibit the PCR, the *Escherichia coli* strain EDL933 DNA was added to each sample as an internal inhibition control (Nielsen and Andersen, 2003) (Supplementary File 2).

### Validation of the BioMark Real-Time PCR Results

Conventional or nested PCRs using primers that targeted different genes or regions than those of the BioMark system were performed on several samples presenting low Ct values or co-infections with multiple pathogen species with individual low Ct values. Each reaction was carried out in a 25 μl reaction volume containing 12.5 μl of 2x Green PCR Mastermix (Rovalab, GmbH, Teltow, Germany), 1 μM of each primer, and 4 μl of DNA sample. The amplification reactions were carried out in C1000 Thermal Cyclers (Bio-Rad, CA, United States), using previously published primers and protocols (Michelet et al., 2014) (**Table 2**).

**TABLE 2:** Primer sets used for pathogen DNA amplification by conventional PCR/nested PCR.

**Pathogen**	**Targeted gene**	**Primer name**	**Sequence**	**Amplicon size (bp)**	**References**
*Borrelia* spp.	*flaB*	FlaLL	5′-ACATATTCAGATGCAGACAGAGGT-3′	664	Barbour et al., 1996; Loh et al., 2017
		FlaRL	5′- TGTTAGACGTTACCGATACTAACG-3′		
		FlaLS	5′ -AACAGCTGAAGAGCTTGGAATG-3′	350	
		FlaRS	5′-CGATAATCTTACTATTCACTAGTTTC-3′		
*Anaplasma phagocytophilum*	*groEL*	EphplgroEL(569)F	5′- ATGGTATGCAGTTTGAT GC-3′	624	Alberti et al., 2005
		EphplgroEL(1193)R	5′- TCTACTCTGTCTTTGCGTTC- 3′		
		EphplgroEL(569)F	5′- ATGGTATGCAGTTTGAT GC-3′	570	
		EphgroEL(1142)R	5′- TTGAGTACAGCAACACCACCGGAA-3′		
*Rickettsia* spp.	*gltA*	Rsfg877	5′-GGG GGC CTG CTC ACG GCG G-3′	381	Regnery et al., 1991
		Rsfg1258	5′- ATT GCA AAA AGT ACA GTG AAC A -3′		
*Bartonella* spp.	*gltA*	bart781	5′-GGG GAC CAG CTC ATG GTG G-3′	380-400	Norman et al., 1995
		bart1137	5′-AAT GCA AAA AGA ACA GTA AAC A-3′		

Amplicons were further processed by sequencing (performed by Macrogen Europe B.V., Amsterdam, Netherlands), for the final confirmation of pathogen species. Thus, the results obtained by the real-time microfluidic PCR assay for 89 *Borrelia* spp., 63 *Anaplasma* spp., 89 *Rickettsia* spp., and 36 *Bartonella* spp. samples were re-tested by conventional or nested PCRs. Following the molecular analysis, 80 *Borrelia* spp., 53 *A. phagocytophilum*, and 44 *Rickettsia* spp. samples were sequenced. Identity percentages of the sequences obtained with reference sequences available in GenBank (NCBI) are presented (Table 3).

**TABLE 3:** Homology between obtained sequences and reference sequences in GenBank.

**Genus**	**No. of tested samples**	**Species obtained after sequencing**	**No. of samples obtained after sequencing**	**Percentage of identity**	**Reference sequence**
*Borrelia*	80	*Bo. afzelii*	43	100	GU826786 (*n* = 17)
				100	MK922620 (*n* = 1)
				100	MF150051 (*n* = 3)
				99	CP018262 (*n* = 11)
				99	MH102392 (*n* = 11)
		*Bo. garinii*	12	100	D89899 (*n* = 5)
				100	KU672556 (*n* = 6)
				99	MK604255 (*n* = 1)
		*Bo. lusitaniae*	9	100	MK604255 (*n* = 5)
				99	MK604255 (*n* = 4)
		*Bo. spielmanii*	5	100	MK604300 (*n* = 5)
		*Bo. valaisiana*	4	100	MK604286 (*n* = 2)
				99	MK604286 (*n* = 1)
				99	CP009117 (*n* = 1)
		*Bo. bavariensis/Bo. garinii*	5	100	CP028872/DQ650333 (*n* = 1)
				99	CP028872/DQ650333 (*n* = 4)
		*Bo. miyamotoi*	2	99	CP044783 (*n* = 2)
*Anaplasma*	53	*A. phagocytophilum*	53	100	MF372791 (*n* = 53)
*Rickettsia*	44	*R. helvetica*	40	100	MF673859 (*n* = 1)
				100	MF6738 (*n* = 16)
				100	KY231199 (*n* = 22)
				99	KY231199 (*n* = 1)
		*R. monacensis*	3	100	JX003686 (*n* = 3)
		*R. aeschlimannii*	1	100	AY259084 (*n* = 1)

The sequences were compared to other GenBank entries by BLAST (Basic Local Alignment Search Tool) analysis and further submitted to the GenBank under the following accession numbers*: Bo. afzelii* (MW272725, MW272726, MW272727, MW272728, MW272729, MW272730, MW272731, MW272732, MW272733, MW272734); *Bo. garinii* (MW272735, MW272736, MW272737, MW272738, MW272739, MW272740); *Bo. lusitaniae* (MW272741, MW272742); *Bo. spielmanii* (MW272743, MW272744); *Bo. valaisiana* (MW272745, MW272746, MW272747); *Bo. bavariensis* (MW272749, MW272750); *Bo. miyamotoi* (MW272748); *A. phagocytophilum* (MW272751, MW272752); *R. helvetica* (MW272753, MW272755, MW272756, MW272757); *R. monacensis* (MW272758); *R. aeschlimannii* (MW272754). Five *Borrelia* spp. samples showcased equal identity percentages with both *Bo. bavariensis* and *Bo. garinii* reference sequences. Since this title formulation is not accepted by GenBank the two individual sequences were submitted as *Bo. bavariensis*.

### Statistical Analysis

Statistical calculations were performed using EpiInfo 7 software (CDC, Atlanta, GE, United States). The prevalence was established and differences between various groups were assessed using chi-square tests. All differences were considered statistically significant for *p* < 0.05.

## Results

By using the microfluidic PCR assay 443 samples were analyzed: 151 questing ticks, 222 engorged ticks, and 70 tissue and blood samples collected from urban wildlife. Overall, by considering the results of both methods used (microfluidic PCR, conventional, and nested PCRs), of the targeted pathogens, 15 were detected to species level, and 6 to genus level in the seven locations in Cluj-Napoca (Supplementary File 1).

### Questing Ticks

#### *Borrelia* spp. in *Ixodes ricinus*

**FIGURE 1:**
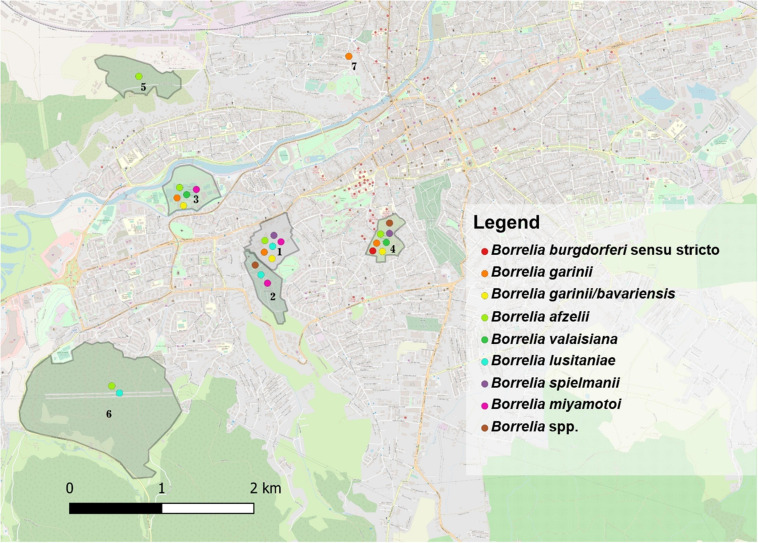
The distribution of *Borrelia* spp. in questing and engorged ticks in the seven locations assessed in Cluj-Napoca. (1) USAMV Campus; (2) Mănăştur Cemetery; (3) Iuliu Haţieganu Park; (4) Alexandru Borza Botanical Garden; (5) Hoia forest; (6) Făget forest; (7) Private garden.

*Borrelia burgdorferi* s.l DNA was detected in 36/95 (37.9%) of questing *I. ricinus* ticks collected from all sampling sites. Infection rates were not significantly different between females 43.8% (14/32); males 36.3% (12/33), and nymphs 33.3% (10/30) (χ^2^ = 1.65; d.f. = 2; *p* = 0.437), or between ticks collected in urban areas compared to peri-urban sites (χ^2^ = 0.5; d.f. = 1; *p* = 0.475). Seven species of *Borrelia* were identified: *Bo. afzelii* (12.6%), *Bo. lusitaniae* (9.5%), *Bo. garinii* (7.4%), *Bo. spielmanii* (3.2%), *Bo. burgdorferi* s.s. (2.1%), *Bo. valaisiana* (2.1%), *Bo. bavariensis/Bo. garinii* (1.05%), and *Bo. miyamotoi* (1.05%), with statistically significant differences among locations only in the case of *I. ricinus* ticks from Mănăştur Cemetery and Făget forest which were more frequently infected with *Bo. lusitaniae* (χ^2^ = 21.10; d.f. = 6; *p* = 0.001). All aforementioned *Borrelia* spp. were detected in ticks in the urban locations, while *Bo. afzelii* and *Bo. lusitaniae* were the only two species present in peri-urban ticks (Figure 1 and Supplementary File 3).

#### Other Tick-Borne Pathogens in *Ixodes ricinus*

*Anaplasma phagocytophilum* DNA had a prevalence of 24.2% (23/95) in questing *I. ricinus* ticks: females 21.9% (7/32), males 33.3% (11/33), nymphs 16.7% (5/30), and was found in ticks collected from all the seven locations, without statistically significant differences among locations (χ^2^ = 4.38; d.f. = 6; *p* = 0.624), or stages (χ^2^ = 2.52; d.f. = 2; *p* = 0.283), except for Iuliu Haţieganu Park, which recorded a significantly higher prevalence of infection in adult ticks: 40% in females (95% CI: 5.27–85.34) and 50% in males (95% CI: 1.26–98.74) (χ^2^ = 6.21; d.f. = 2; *p* = 0.044) compared to the other locations.

**FIGURE 2:**
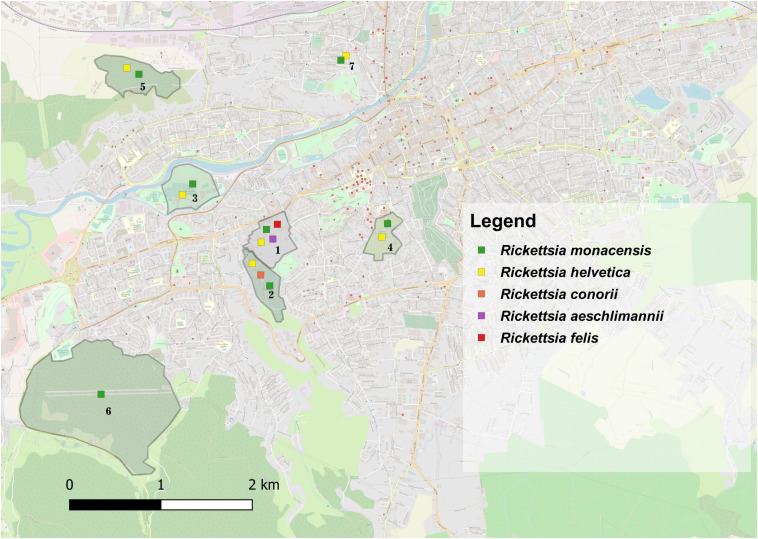
The distribution of *Rickettsia* spp. in questing and engorged ticks in the seven locations assessed in Cluj-Napoca. (1) USAMV Campus; (2) Mănăştur Cemetery; (3) Iuliu Haţieganu Park; (4) Alexandru Borza Botanical Garden; (5) Hoia forest; (6) Făget forest; (7) Private garden.

Two *Rickettsia* species were found in *I. ricinus* ticks. *Rickettsia helvetica* had an overall prevalence of 22.1% (21/95): females 18.75% (6/32), males 18.2% (6/33), nymphs 30% (9/30), without statistical differences among life stages (χ^2^ = 1.59; d.f. = 2; *p* = 0.451). This species was detected only in urban *I. ricinus* ticks (χ^2^ = 4.81; d.f. = 1; p = 0.028). *Rickettsia monacensis* had a prevalence of 12.6% (12/95): females 18.75% (6/32), males 12.1% (4/33), nymphs 6.7% (2/30), without statistical differences among life stages (χ^2^ = 2.06; d.f. = 2; *p* = 0.356), but with a statistically significant difference of the prevalence rate among locations (χ^2^ = 13.16; d.f. = 6; *p* = 0.040), respectively higher for the peri-urban environment (16.7%) (95% CI: 3.58–41.42) (Figure 2 and Supplementary File 3).

#### Tick-Borne Pathogens in Other Tick Species

Three *Borrelia* spp. were found in 3/53 (5.7%) of the *Haemaphysalis punctata* ticks collected in three locations (Iuliu Haţieganu Park, Mănăştur Cemetery, and Hoia forest) as follows: *Bo. afzelii* (1.9%), *Bo. garinii* (1.9%), and *Bo. lusitaniae* (1.9%) (Supplementary File 3). Between locations, the prevalence of infection with *Bo. burgdorferi* s.l was statistically higher in *H. punctata* nymphs than adults (7.14%) (95% CI: 0.88–23.5) (χ^2^ = 15.43; d.f. = 4; *p* = 0.003).

The prevalence of infection with *A. phagocytophilum* in *H. punctata* ticks from Făget forest and Mănăştur Cemetery was 18.9% (10/53): females 9.09% (1/11); males 28.6% (4/14); and nymphs 17.9% (5/28). No statistical differences in prevalence among locations or life stages were recorded.

Three *Rickettsia* spp. were detected in *H. punctata* ticks: *R. monacensis* 15.09% (8/53): females 27.3% (3/11); males 0%; nymphs 17.9% (5/28); *R. helvetica* 13.2% (7/53): females 27.3% (3/11); males 21.4% (3/14); nymphs 3.6% (1/28); and *R. conorii* 1.9%: females 9.09% (1/11). No statistical differences were recorded for the prevalence of infection with Rickettsiales among locations or life stages in *H. punctata* ticks.

The only *D. marginatus* specimen included tested positive for *A. phagocytophilum* DNA. Also, both *D. reticulatus* individuals were positive for *Rickettsia* spp. DNA.

Overall, among the tick species analyzed, the prevalence of *Bo. burgdorferi* s.l. was statistically higher in questing ticks collected in urban areas (30.28%; 95% CI: 21.84–39.81) than peri-urban ones (15.38%; 95% CI: 5.86–30.53) (χ^2^ = 10.82; d.f. = 1; *p* < 0.001).

### Urban Wildlife Hosts

#### *Borrelia* spp. in Engorged Ticks

*Borrelia burgdorferi* s.l. DNA was present in engorged ticks collected from urban wildlife as follows: *I. ricinus* 36.6% (60/164), of which 53.3% (8/15) in females, 46.7% (7/15) in males, 42.6% (26/61) in nymphs, and 26% in larvae (19/73); *I. hexagonus* 60% (21/35) of which 66.7% (18/27) in females and 37.5% (3/8) in males; *H. punctata* 6.25% (1/16) in nymphs. The overall prevalence of infection was 24.8% for *Bo. afzelii* (with statistically significant differences between locations (χ^2^ = 11.21; d.f. = 3; p = 0.01), 4.05% for *B. garinii*, 2.25% for *Bo. spielmanii*, 2.25% for *Bo. valaisiana*, 2.25% for *Bo. bavariensis/Bo. garinii*, and 1.8% for *Bo. miyamotoi*. The prevalence of *Bo. afzelii* was significantly higher in urban *I. hexagonus* (44.4%; 95% CI: 27.94–61.9) compared to *I. ricinus* (24.2; 95% CI: 17.73-31.67) (χ^2^ = 4.99; d.f. = 1; *p* = 0.025). There were statistically significant differences in the urban areas among the prevalence of *Bo. burgdorferi* s.l. between tick species (χ^2^ = 13.37; d.f. = 2; *p* = 0.001), with a significantly higher prevalence in *I. hexagonus* (58.3%; 95% CI: 40.76–74.49). *Borrelia miyamotoi* DNA was found in two co-feeding *I. ricinus* ticks (1 larva and 1 nymph) collected from the same *E. roumanicus* individual. Nevertheless, the blood sample collected from the respective hedgehog was negative for *Bo. miyamotoi* (Figure 1 and Supplementary File 3).

#### Other Tick-Borne Pathogens in Engorged Ticks

*Anaplasma phagocytophilum* DNA had a prevalence of 60.4% (99/164) in *I. ricinus* ticks, of which 66.7% (10/15) in females, 80% (12/15) in males, 68.9% (42/61) in nymphs, and 48% (35/73) in larvae, with significant differences in prevalence between life stages (χ^2^ = 9.2; d.f. = 3; *p* = 0.026). Also, 92.6% (25/27) of females and 100% (8/8) of males of *I. hexagonus*, and 25% (4/16; 2 nymphs and 2 larvae) of *H. punctata* tested positive for the presence of *A. phagocytophilum* DNA. The overall prevalence of *A. phagocytophilum* infection was significantly higher in *I. hexagonus* (91.7%; 95% CI: 77.54–98.25), compared to *I. ricinus* (60.4%; 95% CI: 52.44–67.91) (χ^2^ = 11.53; d.f. = 1; *p* = 0.0006). A percentage of 98.4% (134/136) of the *A. phagocytophilum*-positive engorged ticks were collected from urban hedgehogs. The remaining two ticks were collected from birds (*Phylloscopus collybita* and *Turdus merula*).

*Rickettsia helvetica* DNA was found in 30.5% (50/164) of *I. ricinus* ticks (53.3%, 8/15 females; 53.3% 8/15 males; 29.5%, 18/61 nymphs, and 21.9% 16/73 larvae) and 52.8% (19/36) of *I. hexagonus* ticks (44.4% 12/27 females, and 87.5% 7/8 males) with statistical differences regarding the prevalence of infection between the two species (χ^2^ = 5.54; d.f. = 1; *p* = 0.018), and among locations (χ^2^ = 29.93; d.f. = 3; *p* = 0). *R. monacensis* was the second most prevalent *Rickettsia* spp., detected in 6.1% of *I. ricinus*, 8.3% of *I. hexagonus*, 12.5% of *H. punctata*, and 16.7% of *H. concinna* ticks. Also, in USAMV Campus, one *I. ricinus* nymph tested positive for the presence of *R. felis* and one *H. concinna* nymph for *R. aeschlimannii* DNA (Figure 2). *Hepatozoon* spp. had a 0.9% prevalence in the engorged ticks analyzed, in which we also detected *Theileria* spp., with a 2.25% prevalence (Supplementary File 3).

### Urban Wildlife Tissue Samples

#### Rodents

Rodents were considered positive if pathogenic DNA was detected in any of the tissue samples collected. Therefore, three *Borrelia* spp. (*Bo. afzelii*, *Bo. spielmanii*, and *Bo. miyamotoi*) were found in 13.8% (4/29) of rodents, as follows: the skin biopsy and heart tissue of one *Arvicola terrestris* from USAMV Campus, and the skin biopsy of one *Apodemus agrarius* from Iuliu Haţieganu Park were positive for *Bo. afzelii*, while both *Bo. miyamotoi*, and *Bo. spielmanii* DNA were detected in the skin biopsy of two different *A. agrarius* (one pathogen/rodent) from Iuliu Haţieganu Park. *Anaplasma phagocytophilum* DNA was detected in the heart tissue of one *A. flavicollis*. Also, the prevalence of *R. monacensis* was 17.2% (5/29), detected individually in the skin biopsies of three *A. agrarius* and one *Apodemus sylvaticus* from USAMV Campus, and one *Mus musculus* from Hoia forest, while *N. mikurensis* had a prevalence of 2.9% (2/29) in the skin biopsy and liver tissue of one *A. terrestris* from USAMV Campus, and in the heart and liver tissue of one *A. agrarius* from Iuliu Haţieganu Park (Supplementary File 1).

#### Birds

*Borrelia afzelii* was detected in the blood sample of one urban *Parus major* 3% (1/33). Also, 30.3% (10/33) of urban birds [*Corvus frugilegus* (*n* = 1), *Erithacus rubecula* (*n* = 1), *Garrulus glandarius* (*n* = 1), *Parus major* (*n* = 3), *Sturnus vulgaris* (*n* = 1), and *Turdus merula* (*n* = 3)] harbored *A. phagocytophilum* DNA, 12.1% (4/33) *R. helvetica* DNA [*Corvus frugilegus* (*n* = 1), and *Parus major* (*n* = 3)], and 3% (1/33) *R. monacensis* DNA [*Turdus merula* (*n* = 1)] (Supplementary File 1).

#### Hedgehogs

Of the eight blood samples tested, two were positive for *A. phagocytophilum* DNA, and one for *R. helvetica* DNA.

### Co-infections Between Tick-Borne Pathogens

#### Questing Ticks

##### Ixodes ricinus

**FIGURE 3:**
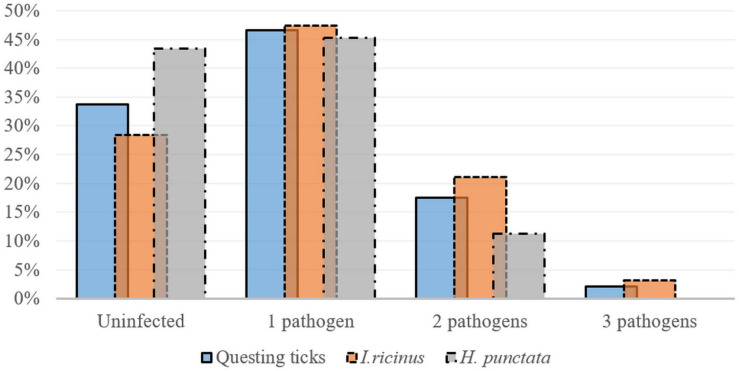
Single and multiple infections (percent of total collected ticks) detected in questing *Ixodes ricinus* (*n* = 95) and *H.* punctata (*n* = 53) ticks collected in Cluj-Napoca.

Co-infections occurred in 34.3% (23/67) of all *I. ricinus* infected ticks. Co-infection prevalence was 36% (9/25) in females, 29.2% (7/24) in males and 38.9% (7/18) in nymphs. A statistically significant difference was recorded regarding the prevalence of co-infections among life stages in the peri-urban sites: 0% in females; 25% (95% CI: 0.63–80.59) in males; and 100% (95% CI: 15.81–100) in nymphs (χ^2^ = 7.21; d.f. = 2; *p* = 0.027). The most frequent dual co-infections were between *Rickettsia* spp. and *Borrelia* spp., followed by *Rickettsia* spp. and *A. phagocytophilum*, and *A. phagocytophilum* and *Borrelia* spp. (Figure 3).

Co-infections with three pathogens were less common and consisted of combinations between *R. helvetica*, *A. phagocytophilum*, and *Borrelia*. spp. (Table 4).

**TABLE 4:** Co-infections with tick-borne pathogens in questing ticks in Cluj-Napoca.

**Location**	**Environment**	**Tick species**	**Percentage of co-infected ticks from total questing ticks/location/species**	**Life stage (no. of specimens)**	**Pathogen species**
USAMV Campus	Urban	*I. ricinus*	5.8 (*n* = 17)	F (*n* = 1)	*A.ph* + *Bo.g*
USAMV Campus	Urban	*I. ricinus*	11.8 (*n* = 17)	F (*n* = 2)	*R.h* + *Bo.l*
USAMV Campus	Urban	*I. ricinus*	5.8 (*n* = 17)	F (*n* = 1)	*R.h* + *Bo.s*
Mănăştur Cemetery	Urban	*I. ricinus*	8.3 (*n* = 12)	F (*n* = 1)	*A.ph* + *Bo.l*
Mănăştur Cemetery	Urban	*I. ricinus*	25 (*n* = 12)	M (*n* = 2) F (*n* = 1)	*R.m* + *Bo.l*
Mănăştur Cemetery	Urban	*I. ricinus*	8.3 (*n* = 12)	M (*n* = 1)	*R.h* + *A.ph* + *Bo.m*
Mănăştur Cemetery	Urban	*H. punctata*	5.8 (*n* = 17)	F (*n* = 1)	*R.c* + *Bo.l*
Mănăştur Cemetery	Urban	*H. punctata*	11.8 (*n* = 17)	F (*n* = 2)	*R.h* + *Borrelia* spp.
Mănăştur Cemetery	Urban	*H. punctata*	5.8 (*n* = 17)	F (*n* = 1)	*R.m* + *Borrelia* spp.
Iuliu Haţieganu Park	Urban	*I. ricinus*	5.3 (*n* = 19)	F (*n* = 1)	*A.ph* + *Bo.a*
Iuliu Haţieganu Park	Urban	*I. ricinus*	5.3 (*n* = 19)	N (*n* = 1)	*R.h* + *Bo.g*
Iuliu Haţieganu Park	Urban	*I. ricinus*	5.3 (*n* = 19)	N (*n* = 1)	*R.m* + *Bo.v*
Alexandru Borza Botanical Garden	Urban	*I. ricinus*	13.6 (*n* = 22)	F (*n* = 1) M (*n* = 2)	*R.h* + *A.ph*
Alexandru Borza Botanical Garden	Urban	*I. ricinus*	4.5 (*n* = 22)	F (*n* = 1)	*R.h* + *Bo.v*
Alexandru Borza Botanical Garden	Urban	*I. ricinus*	4.5 (*n* = 22)	N (*n* = 1)	*R.h* + *A.ph* + *Bo.a*
Alexandru Borza Botanical Garden	Urban	*I. ricinus*	4.5 (*n* = 22)	N (*n* = 1)	*R.h* + *A.ph* + *Bo.b ss*
Hoia forest	Peri-urban	*I. ricinus*	7.7 (*n* = 13)	N (*n* = 1)	*A.ph* + *Theileria* spp.
Făget forest	Peri-urban	*I. ricinus*	20 (*n* = 5)	N (*n* = 1)	*A.ph* + *Theileria* spp.
Făget forest	Peri-urban	*I. ricinus*	20 (*n* = 5)	M (*n* = 1)	*R.m* + *A.ph*
Făget forest	Peri-urban	*H. punctata*	10 (*n* = 20)	N (*n* = 2)	*R.m* + *A.ph*
Private garden	Urban	*I. ricinus*	14.3 (*n* = 7)	M (*n* = 1)	*A.ph* + *Bo.g*
Private garden	Urban	*I. ricinus*	14.3 (*n* = 7)	N (*n* = 1)	*R.m* + *A.ph*

*F, female; M, male; N, nymph; *A. ph, Anaplasma phagocytophilum; Bo.a, Borrelia afzelii; Bo.g, Borrelia garinii; Bo.m, Borrelia miyamotoi; Bo.b ss, Borrelia burgdorferi* sensu stricto*; Bo.s, Borrelia spielmanii; Bo.v, Borrelia valaisiana; Bo.l, Borrelia lusitaniae; R.h, Rickettsia helvetica; R.m, Rickettsia monacensis; R.c, Rickettsia conorii.**

##### Haemaphysalis punctata

Co-infections were present in 20% (6/30) of all infected *H. punctata* ticks. Co-infection prevalence was 50% (4/8) in females, and 13.3% (2/15) in nymphs, with a statistically higher prevalence of infection in females 57.14% (95% CI: 18.41–90.1), compared to nymphs (0% prevalence) in urban areas (χ^2^ = 6.85; d.f. = 2; *p* = 0.032). The most prevalent co-infections were between *Rickettsia* spp. and *Borrelia* spp., followed by *Rickettsia* spp. and *A. phagocytophilum* (Table 4 and Figure 3). One *D. reticulatus* from Făget forest was co-infected with *Borrelia* spp. and *Rickettsia* spp.

Globally, there were no statistically significant correlations between the co-infection rates in questing ticks and the environment (urban/peri-urban) (χ^2^ = 0.49; d.f. = 1; *p* = 0.483). Despite the lack of other significant correlations, the urban areas showed a more diverse array of pathogen species compared to the peri-urban sites (Figures 1, 2).

#### Engorged Ticks

Of the engorged infected ticks, 69.2% (108/156) were co-infected with various TBPs.

##### *Ixodes spp*.

**FIGURE 4:**
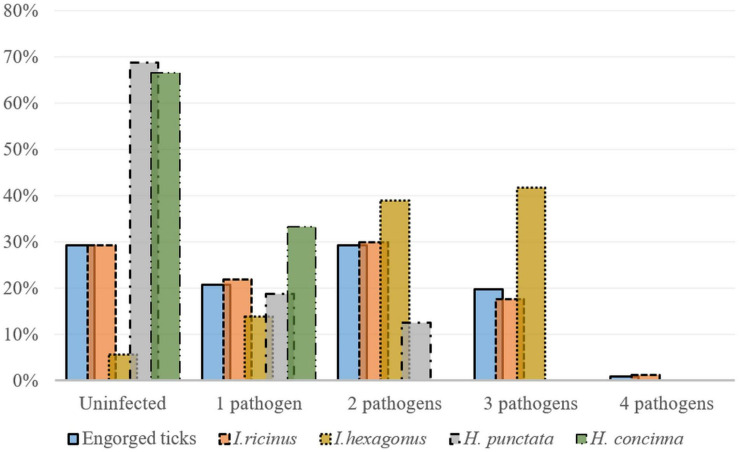
Co-infections detected in engorged *Ixodes ricinus* (*n* = 164), *I. hexagonus* (*n* = 36), *Haemaphysalis punctata* (*n* = 16), and *H. concinna* (*n* = 6) ticks (percent of total collected ticks) collected from wildlife hosts in Cluj-Napoca.

From the total infected *I. ricinus* ticks, 69% (79/115) were co-infected with multiple pathogens. Of these 72.7% (8/11) were females, 91.7% (11/12) males, 66% (33/50) nymphs and 65.1% (27/42) were larvae. Of the *I. hexagonus* analyzed, 79.4% (27/34) were co-infected: 76.9% (20/26) were females, and 87.5% (7/8) males (Figure 4). The most frequent co-infections in *Ixodes* spp. were dual co-infections equally prevalent with *A. phagocytophilum* and *Borrelia* spp., and *A. phagocytophilum* and *R. helvetica*, followed by infections with three pathogens namely *R. helvetica*, *A. phagocytophilum*, and *Borrelia* spp. (Supplementary File 4).

##### *Haemaphysalis* spp.

One *H. punctata* showed a co-infection with *A. phagocytophilum* and *Bo. afzelii*, while another one was co-infected with *A. phagocytophilum* and *R. monacensis*.

All the co-infected engorged ticks were collected from hosts found in urban locations (Figure 4 and Supplementary File 4). Overall, there were significant differences among the engorged tick species between locations in terms of co-infection rates (χ^2^ = 8.6; d.f. = 2; *p* = 0.013).

#### Urban Wildlife Tissue Samples

Co-infections were also detected in two tissue samples collected from rodents. One *A. agrarius* from Iuliu Haţieganu Park tested positive for *Bo. miyamotoi* and *N. mikurensis*, and one *A. terrestris* from USAMV Campus was co-infected with *Bo. afzelii* and *N. mikurensis*. Also, three samples collected from birds (one *Parus major* and one *Turdus merula* from Alexandru Borza Botanical Graden, and one *Corvus frugilegus* from Iuliu Haţieganu Park) presented dual co-infections with *A. phagocytophilum* and *Rickettsia* spp., while one sample (*Parus major* from Alexandru Borza Botanical Garden) showed a triple co-infection with *A. phagocytophilum*, *R. helvetica, and Bo. afzelii* (Table 5).

**TABLE 5:** Co-infections with tick-borne pathogens in tissue samples collected from urban wildlife hosts in Cluj-Napoca.

**Location**	**Host species**	**Environment**	**Co-infection**
USAMV Campus	*Arvicola terrestris*	Urban	*N.m* + *Bo.a*
Iuliu Haţieganu Park	*Apodemus agrarius*	Urban	*N.m* + *Bo.m*
Iuliu Haţieganu Park	*Corvus frugilegus*	Urban	*A.ph* + *R.h*
Alexandru Borza Botanical Garden	*Turdus merula*	Urban	*A.ph* + *R.m*
Alexandru Borza Botanical Garden	*Parus major*	Urban	*A.ph* + *R.h*
Alexandru Borza Botanical Garden	*Parus major*	Urban	*A.ph* + *R.h* + *Bo.a*

**A.ph, Anaplasma phagocytophilum; Bo.a, Borrelia afzelii; Bo.m, Borrelia miyamotoi; R.h, Rickettsia helvetica; R.m, Rickettsia monacensis; N.m, Neoehrlichia mikurensis.**

## Discussion

Using the BioMark system we performed a comprehensive survey of the various TBPs that co-circulate in tick-host cycles in five urban, and two peri-urban locations in Cluj-Napoca, a major city in Romania. Even though local prevalence studies have limited value in terms of epidemiological risk assessment, the prevalence of *Bo. burgdorferi* s.l. spirochetes in questing ticks has been considered an essential element of risk assessment for Lyme borreliosis (LB) (Rauter and Hartung, 2005). Therefore, collectively, the results of this study may have important implications in terms of public health, especially for the urban areas, since until recently LB risk was considered to be correlated with residency in rural areas (Rizzoli et al., 2011).

Nowadays, a higher LB incidence is registered in urban environments (3.2%/100.000 inhabitants) than in rural settlements (2.5%/100.000 inhabitants) in Romania (NCSCC, 2018). Increased access to information and better accessibility and addressability to medical services of the urban population could explain the more common reference of patients to hospitals for diagnostic purposes, hence, the higher incidence (NCSCC, 2018).

The overall *Borrelia burgdorferi* s.l. prevalence (37.9%) in questing *I. ricinus* ticks across all locations assessed in this study was much higher compared to previous data on the prevalence of Lyme spirochetes reported in questing ticks from Romania by conventional PCR studies (3.8–18%) (Coipan and Vladimirescu, 2011; Kalmár et al., 2013), but similar to the prevalence reported in Iaşi county (25.8%) by microfluidic real-time PCR (Raileanu et al., 2017).

Since some clinical manifestations are specific to particular *Borrelia* species, their prevalence in a certain area is important for risk assessment (Strnad et al., 2017). As in our study, a Europe-wide meta-analysis of *Bo. burgdorferi* s.l. species in questing ticks (Estrada-Peña et al., 2018) and previous reports in Romania (Kalmár et al., 2013) showed that the most prevalent *Borrelia* spp. are *Bo. afzelii*, and *Bo. garinii*.

Accounting for most of the LB human cases in Europe, *Bo. afzelii* is mostly isolated from medium-sized and small rodents (Coipan et al., 2018). *Borrelia garinii* is commonly hosted by birds (Dubska et al., 2009), particularly species that can reach high densities in urban sites (Taragel’ová et al., 2008). Nevertheless, a distinct and highly pathogenic ecotype of *Bo. garinii*, now confirmed to species status, *Bo. bavariensis* (formerly known as OspA type 4) uses rodents as reservoir hosts (Huegli et al., 2002; Margos et al., 2013). The relatively high prevalence of these *Borrelia* spp. could be linked to the high diversity and abundance of rodent species in Romania (Mihalca et al., 2012b). Commonly associated with birds (Rizzoli et al., 2011), *Bo. valaisiana* had a lower prevalence in questing ticks from Cluj-Napoca than in Iaşi county (Raileanu et al., 2017) and was only present in urban locations.

We report higher *Bo. lusitaniae* infection rates in questing ticks in Cluj-Napoca compared to previous studies in Romania (Kalmár et al., 2013; Raileanu et al., 2017). *Borrelia lusitaniae* is mainly associated with lizards (Rizzoli et al., 2011). Heltai et al. (Heltai et al., 2015) reported that cemeteries contribute significantly to the habitats of lizards in urban areas due to the presence of stony habitats, their size, heterogeneity, and reduced levels of human disturbance. Thus, the significantly higher prevalence of infection with *Bo. lusitaniae* of questing ticks from Mănăştur Cemetery is most likely linked to the presence and abundance of lizards (confirmed through visual inspection - data not shown) in the respective site.

*Borrelia miyamotoi*, the only relapsing fever agent transmitted by *Ixodes* species in Europe (Cutler et al., 2019), was initially reported in questing ticks in central Romania (Kalmár et al., 2016). Recently, Raileanu et al. confirmed the low infection rate in ticks from eastern Romania (Raileanu et al., 2017). We also confirm the presence of *Bo. miyamotoi* at a low prevalence in ticks and wildlife hosts in recreational areas of Cluj-Napoca.

*Borrelia* spp. infection and co-infection rates were not significantly different between adults and nymphs of questing *I. ricinus* ticks. The prevalence of *Bo. burgdorferi* s.l. in Europe is higher in adults than in nymphs (Strnad et al., 2017). Nonetheless, nymphs are mainly responsible for transmitting *Borrelia* spp. to humans (Rizzoli et al., 2011) and can be encountered in suburban and urban environments (Pejchalová et al., 2007), and even roadsides (Haemig et al., 2008).

The public health relevance of *H. punctata* ticks is considered to be rather limited (Briciu et al., 2014). Moreover, its vectorial role for *Borrelia* spp. has not been clearly demonstrated. As in our study, LB spirochetes have formerly been reported in questing *H. punctata* (Tälleklint, 1996), but at lower prevalence compared to *I. ricinus*.

The presence of *A. phagocytophilum* has been investigated in detail in Romania (Matei et al., 2015; Kalmár et al., 2016; Raileanu et al., 2017; Raileanu et al., 2018). The higher prevalence of *A. phagocytophilum* infection in adult *I. ricinus* compared to nymphs could be linked to the greater number of bloodmeals, since transstadial transmission of *A. phagocytophilum* is improbable (Raileanu et al., 2018). Therefore, despite no reports of human infection with *A. phagocytophilum* in Romania so far, the risk of acquiring this pathogen following tick bites in recreational areas is possible.

In the current study, the dominant SFG *Rickettsia* species in questing ticks from both urban and peri-urban areas were *R. helvetica* and *R. monacensis*, in concordance with former mentions in urban sites in Romania (Raileanu et al., 2018), and Europe (Rizzoli et al., 2014; Kowalec et al., 2019). Here we report for the first time the presence of *R. aeschlimannii* in Romania, identified in *H. concinna*. *Rickettsia aeschlimannii* is an emerging human and animal pathogen, reported from various ticks in Europe and Africa, including several *Hyalomma* spp. ticks collected from migrant bird species (Parola et al., 2013; Chisu et al., 2016). Another SFG *Rickettsia* present in this study is *R. felis* which is known to be transmitted via cat flea bites (Brown and Macaluso, 2016). Despite seldom reports regarding the presence and prevalence of this TBP, several other studies have also identified *R. felis* in questing *I. ricinus* ticks in Europe (Vayssier-Taussat et al., 2013; Lejal et al., 2019). A previous study in Italy (Ciervo et al., 2006) also reports the presence of the human pathogen *R. conorii* in *H. punctata* questing ticks. However, further research is required to assess the vectorial competence of *Haemaphysalis* spp. and *I. ricinus* and their implication in the transmission of these SFG *Rickettsia* spp.

Nowadays, thanks to more sensitive and efficient detection tools, co-infections with different TBPs are more frequently reported in ticks (Michelet et al., 2014; Raileanu et al., 2017; Lejal et al., 2019; Gondard et al., 2020). The pathogenesis and aftermath of co-infections in humans is a complex process that still needs further research (Baneth, 2014). Pathogens can synergistically colonize more favorably their hosts through processes initiated by co-transmission and entering of multiple pathogens inside the respective host’s organism (Baneth, 2014).

We report a high prevalence of co-infections in both questing and engorged ticks. Contrary to previous reports which mentioned *Bo. afzelii* and *Bo. garinii* as the most prevalent co-infection in ticks in Romania (Raileanu et al., 2017), or France (Moutailler et al., 2016), we hereby detected co-infections among *Rickettsia* spp. and *Borrelia* spp., followed by *Rickettsia* spp. and *A. phagocytophilum*, and *A. phagocytophilum* and *Borrelia* spp., as most prevalent. A high prevalence of co-infection with *R. helvetica* and *A. phagocytophilum* in *I. ricinus* ticks was also reported by Lejal et al. (Lejal et al., 2019) who suggested that the superior acclimatization of these two pathogens in ticks might portend them as stronger competitors than other pathogen species. Our findings also uncover new risks for urban inhabitants since the co-infection with *A. phagocytophilum* and *Bo. burgdorferi* s.l. was shown to enhance the colonization ability of *Bo. burgdorferi* s.l. (Grab et al., 2007).

Despite our statistical analysis revealing a significantly higher prevalence of pathogens in urban ticks compared to the peri-urban ones, the sample sizes assessed were uneven, as the majority of samples were collected from urban sites. This was not related to bias in the selection algorithm but because of the availability and abundance of wildlife hosts and questing ticks when the collection was performed. A previous study we conducted in these seven recreational locations in Cluj-Napoca showed a higher abundance of ticks in the urban versus the peri-urban locations, linked to the abundance and diversity of local wildlife species, particularly hedgehogs (*E. roumanicus*) (Borşan et al., 2020). These findings may explain the more diverse TBPs community we detected in urban sites, further highlighting the importance of urban dwellers such as hedgehogs, rodents, and birds in the ecology of tick-borne diseases. Given the results of the present study, *E. roumanicus* could facilitate pathogen exchange among infected and uninfected ticks without displaying a systemic infection (through co-feeding mechanisms, pathogens stationed in tissues rather than in the bloodstream) (Randolph, 2011; Voordouw, 2015), and therefore can be considered an amplifier host and an epidemiologically important wildlife species for the urban environment (Jahfari et al., 2017).

Since *I. ricinus* ticks have a high affinity for biting humans and the level of co-infections detected in this tick species in Cluj-Napoca is high, co-transmission and enhanced disease severity in humans are possible scenarios for the city inhabitants (Moutailler et al., 2016).

## Conclusion

The most noteworthy outcomes of this study are (1) the detection of a high prevalence of *Bo. burgdorferi* s.l. in urban questing ticks; (2) the overall great diversity and prevalence of TBPs in engorged ticks collected from urban sites (3) co-infections were frequent in both questing and engorged ticks.

Therefore, additional tick-surveillance and awareness programs should be implemented, especially in recreational areas, since the TBPs detected in ticks in Cluj-Napoca pose a significant risk to human health.

## Data Availability Statement

The datasets presented in this study can be found in online repositories. The names of the repository/repositories and accession number(s) can be found in the article/ Supplementary Material.

## Ethics Statement

The flagging, sampling, and trapping campaigns were performed with the consent of Cluj-Napoca City Hall (Technical Department: Decision No. 13345/442/18.01.2018 and Urban Ecology and Green Spaces Department: Decision No. 13.351/10.01.2018) and the support of the Romanian Ornithological Society (SOR), Babeş Bolyai University (Decision No. 210/090/2018), the University of Agricultural Sciences and Veterinary Medicine of Cluj-Napoca and the owners of the private garden. All the activities were performed according to ethical permits and national legislation.

## Author Contributions

AM and S-DB conceptualized the study. S-DB, AT-N, and AS performed the field work. CP executed the hedgehog anesthesia and sampling. S-DB performed the necropsy examination of the rodents, morphological identification of the ticks, and wrote the first draft of the manuscript. S-DB and AI executed the laboratory work and data analysis. AI implemented the statistical analysis of results and designed the maps. CG and SM designed the microfluidic PCR protocol, processed the samples, and interpreted the results. AM reviewed the manuscript for important intellectual content. All authors have read and approved the final manuscript.

## Conflict of Interest

The authors declare that the research was conducted in the absence of any commercial or financial relationships that could be construed as a potential conflict of interest.
