# Supplementary material for: High Diversity, Prevalence, and Co-infection Rates of Tick-Borne Pathogens in Ticks and Wildlife Hosts in an Urban Area in Romania
Source: Front Microbiol. 2021 Mar 9;12:645002. doi: 10.3389/fmicb.2021.645002 (PMC7985354; doi:10.3389/fmicb.2021.645002)
Supplement: Supplementary file 2 [file Table_2.docx]

**S2.** List of primer sets for DNA amplification used for the BioMark system. Primers were designed by Michelet et al. (Michelet et al., 2014), Gondard et al. (Gondard et al., 2020), and Sprong et al. (Sprong et al., 2019).

| **Pathogen** | **Target gene** | **Primers** | **Nucleotide sequence (5**^′^ **–3**^′^ **)** | **Amplicon** |
| --- | --- | --- | --- | --- |
|  |  |  |  | **size (bp)** |
| *Borrelia burgdorferi* sensu stricto | *rpoB* | Bo_bu_rpoB_F | GCTTACTCACAAAAGGCGTCTT | 83 |
|  |  | Bo_bu_rpoB_R, | GCACATCTCTTACTTCAAATCCT |  |
|  |  | Bo_bu_rpoB_P | AATGCTCTTGGACCAGGAGGACTTTCA |  |
| *Borrelia garinii* | *rpoB* | Bo_ga_rpoB_F | TGGCCGAACTTACCCACAAAA | 88 |
|  |  | Bo_ga_rpoB_R | ACATCTCTTACTTCAAATCCTGC |  |
|  |  | Bo_ga_rpoB_P | TCTATCTCTTGAAAGTCCCCCTGGTCC |  |
| *Borrelia afzelii* | *Fla* | Bo_af_fla_F | GGAGCAAATCAAGATGAAGCAAT | 116 |
|  |  | Bo_af_fla_R | TGAGCACCCTCTTGAACAGG |  |
|  |  | Bo_af_fla_P | TGCAGCCTGAGCAGCTTGAGCTCC |  |
| *Borrelia valaisiana* | *ospA* | Bo_va_ospA_F | ACTCACAAATGACAGATGCTGAA | 135 |
|  |  | Bo_va_ospA_R | GCTTGCTTAAAGTAACAGTACCT |  |
|  |  | Bo_va_ospA_P | TCCGCCTACAAGATTTCCTGGAAGCTT |  |
| *Borrelia lusitaniae* | *rpoB* | Bo_lus_rpoB_F | CGAACTTACTCATAAAAGGCGTC | 87 |
|  |  | Bo_lus_rpoB_R | TGGACGTCTCTTACTTCAAATCC |  |
|  |  | Bo_lus_rpoB_P | TTAATGCTCTCGGGCCTGGGGGACT |  |
| *Borrelia spielmanii* | *fla* | Bo_spi_fla_F | ATCTATTTTCTGGTGAGGGAGC | 71 |
|  |  | Bo_spi_fla_R | TCCTTCTTGTTGAGCACCTTC |  |
|  |  | Bo_spi_fla_P | TTGAACAGGCGCAGTCTGAGCAGCTT |  |
| *Borrelia bissettii* | *rpoB* | Bo_bi_rpoB_F | GCAACCAGTCAGCTTTCACAG | 118 |
|  |  | Bo_bi_rpoB_R | CAAATCCTGCCCTATCCCTTG |  |
|  |  | Bo_bi_rpoB_P | AAAGTCCTCCCGGCCCAAGAGCATTAA |  |
| *Borrelia mayonii* | *fla* | Bo_mayo_fla_F | ACA CAC CAT CAT CAC TTT CAG G |  |
|  |  | Bo_mayo_fla_R | TTG AGC ACC TTC TTG AAC AGG |  |
|  |  | Bo_mayo_fla_P | CGC AAC CTG AGC AGT TTG AGT TCC CT |  |
| *Borrelia bavariensis* | *pyrG* | Bo_bavar_pyrG_F | GTC TTT GGT TCA TGT TGG AGC |  |
|  |  | Bo_bavar_pyrG_R | CCC TCA TAT CCT TTG CCT CC |  |
|  |  | Bo_bavar_pyrG_P | AAA GCC TCC GGG AAC AAT AAT GCC GTC AA |  |
| *Borrelia miyamotoi* | *glpQ* | B_miya_glpQ_F | CACGACCCAGAAATTGACACA | 94 |
|  |  | B_miya_glpQ_R | GTGTGAAGTCAGTGGCGTAAT |  |
|  |  | B_miya_glpQ_P | TCGTCCGTTTTCTCTAGCTCGATTGGG |  |
| *Borrelia* spp. | *23S rRNA* | Bo_bu_sl_23S_F | GAGTCTTAAAAGGGCGATTTAGT | 73 |
|  |  | Bo_bu_sl_23S_R | CTTCAGCCTGGCCATAAATAG |  |
| *Anaplasma marginale* | *msp1b* | An_ma_msp1_F | CAGGCTTCAAGCGTACAGTG | 85 |
|  |  | An_ma_msp1_R | GATATCTGTGCCTGGCCTTC |  |
|  |  | An_ma_msp1_P | ATGAAAGCCTGGAGATGTTAGACCGAG |  |
| *Anaplasma platys* | groEL | An_pla_groEL_F | TTCTGCCGATCCTTGAAAACG | 75 |
|  |  | An_pla_groEL_R | CTTCTCCTTCTACATCCTCAG |  |
|  |  | An_pla_groEL_P | TTGCTAGATCCGGCAGGCCTCTGC |  |
| *Anaplasma phagocytophilum* | *msp2* | An_ph_msp2_F | GCTATGGAAGGCAGTGTTGG | 77 |
|  |  | An_ph_msp2_R | GTCTTGAAGCGCTCGTAACC |  |
|  |  | An_ph_msp2_P | AATCTCAAGCTCAACCCTGGCACCAC |  |
| *Anaplasma ovis* | *ovismsp4* | An_ov_msp4_FT | CATTCGACATGCGTGAGTCA | 92 |
|  |  | An_ov_msp4_RT | TTGCTGGCGCACTCACATC |  |
|  |  | An_ov_msp4_PA | GCAGAGAGACCTCGTATGTTAGAGGC |  |
| *Anaplasma centrale* | *groEL* | An_cen_groEL_F | AGCTGCCCTGCTATACACG | 79 |
|  |  | An_cen_groEL_R | GATGTTGATGCCCAATTGCTC |  |
|  |  | An_cen_groEL_P | CTTGCATCTCTAGACGAGGTAAAGGGG |  |
| *Anaplasma bovis* | groEL | An_bo_groEL_F | GGGAGATAGTACACATCCTTG | 73 |
|  |  | An_bo_groEL_R | CTGATAGCTACAGTTAAGCCC |  |
|  |  | An_bo_groEL_P | AGGTGCTGTTGGATGTACTGCTGGACC |  |
| *Anaplasma* spp. | 16S rRNA | Ana_spp_16S_F | CTTAGGGTTGTAAAACTCTTTCAG | 160 |
|  |  | Ana_spp_16S_R | CTTTAACTTACCAAACCGCCTAC |  |
|  |  | Ana_spp_16S_P | ATGCCCTTTACGCCCAATAATTCCGAACA |  |
| *Ehrlichia* spp. | 16SrRNA | Neo_mik_16S_F | GCAACGCGAAAAACCTTACCA | 98 |
|  |  | Neo_mik_16S_R | AGCCATGCAGCACCTGTGT |  |
|  |  | Neo_mik_16S_P | AAGGTCCAGCCAAACTGACTCTTCCG |  |
| *Ehrlichia canis* | gltA | Eh_ca_gltA_F | GACCAAGCAGTTGATAAAGATGG | 136 |
|  |  | Eh_ca_gltA_R | CACTATAAGACAATCCATGATTAGG |  |
|  |  | Eh_ca_gltA_P | ATTAAAACATCCTAAGATAGCAGTGGCTAAGG |  |
| *Neoehrlichia mikurensis* | *groEL* | Neo_mik_groEL_F | AGAGACATCATTCGCATTTTGGA | 96 |
|  |  | Neo_mik_groEL_R | TTCCGGTGTACCATAAGGCTT |  |
|  |  | Neo_mik_groEL_P | AGATGCTGTTGGATGTACTGCTGGACC |  |
| *Rickettsia conorii* | 23S-5S ITS | Ri_co_ITS_F | CTCACAAAGTTATCAGGTTAAATAG | 118 |
|  |  | Ri_co_ITS_R | CGATACTCAGCAAAATAATTCTCG |  |
|  |  | Ri_co_ITS_P | CTGGATATCGTGGCAGGGCTACAGTAT |  |
| *Rickettsia slovaca* | 23S-5S ITS | Ri_slo_ITS_F | GTATCTACTCACAAAGTTATCAGG | 138 |
|  |  | Ri_slo_ITS_R | CTTAACTTTTACTACAATACTCAGC |  |
|  |  | Ri_slo_ITS_P | TAATTTTCGCTGGATATCGTGGCAGGG |  |
| *Rickettsia massiliae* | 23S-5S ITS | Ri_ma_ITS_F | GTTATTGCATCACTAATGTTATACTG | 128 |
|  |  | Ri_ma_ITS_R | GTTAATGTTGTTGCACGACTCAA |  |
|  |  | Ri_ma_ITS_P | TAGCCCCGCCACGATATCTAGCAAAAA |  |
| *Rickettsia helvetica* | 23S-5S ITS | Ri_he_ITS_F | AGAACCGTAGCGTACACTTAG | 79 |
|  |  | Ri_he_ITS_R | GAAAACCCTACTTCTAGGGGT |  |
|  |  | Ri_he_ITS_P | TACGTGAGGATTTGAGTACCGGATCGA |  |
| *Rickettsia aeschlimannii* | ITS | Rick_aesch_ITS_F | CTCACAAAGTTATCAGGTTAAATAG | 134 |
|  |  | Rick_aesch_ITS_R | CTTAACTTTTACTACGATACTTAGCA |  |
|  |  | Rick_aesch_ITS_P | TAATTTTTGCTGGATATCGTGGCGGGG |  |
| *Rickettsia felis* | orfB | Ri_fel_orfB_F | ACCCTTTTCGTAACGCTTTGC | 163 |
|  |  | Ri_fel_orfB_R | TATACTTAATGCTGGGCTAAACC |  |
|  |  | Ri_fel_orfB_P | AGGGAAACCTGGACTCCATATTCAAAAGAG |  |
| *Rickettsia* spp*.* | gltA | Rick_spp_gltA_F | GTCGCAAATGTTCACGGTACTT | 78 |
|  |  | Rick_spp_gltA_R | TCTTCGTGCATTTCTTTCCATTG |  |
|  |  | Rick_spp_gltA_P | TGCAATAGCAAGAACCGTAGGCTGGATG |  |
| *Bartonella henselae* | *Pap31* | Bar_he_pap31_F | CCGCTGATCGCATTATGCCT | 107 |
|  |  | Bar_he_pap31_R | AGCGATTTCTGCATCATCTGCT |  |
|  |  | Bar_he_pap31_P | ATGTTGCTGGTGGTGTTTCCTATGCAC |  |
| *Bartonella* spp. | ssrA | Bart_spp_ssrA_F | CGTTATCGGGCTAAATGAGTAG | 118 |
|  |  | Bart_spp_ssrA_R | ACCCCGCTTAAACCTGCGA |  |
|  |  | Bart_spp_ssrA_P | TTGCAAATGACAACTATGCGGAAGCACGTC |  |
| *Francisella tularensis* and *Francisella-like* endosymbionts | tul4 | Fr_tu_tul4_F | ACCCACAAGGAAGTGTAAGATTA | 76 |
|  |  | Fr_tu_tul4_R | GTAATTGGGAAGCTTGTATCATG |  |
|  |  | Fr_tu_tul4_P | AATGGCAGGCTCCAGAAGGTTCTAAGT |  |
|  | fop4 | Fr_tu_fopA_F | GGCAAATCTAGCAGGTCAAGC | 91 |
|  |  | Fr_tu_fopA_R | CAACACTTGCTTGAACATTTCTAG |  |
|  |  | Fr_tu_fopA_P | AACAGGTGCTTGGGATGTGGGTGGTG |  |
| *Coxiella burnetii* and *Coxiella*-like | idc | Co_bu_icd_F | AGGCCCGTCCGTTATTTTACG | 74 |
|  |  | Co_bu_icd_R | CGGAAAATCACCATATTCACCTT |  |
|  |  | Co_bu_icd_P | TTCAGGCGTTTTGACCGGGCTTGGC |  |
|  | IS111 | Co_bu_IS_F | TGGAGGAGCGAACCATTGGT | 86 |
|  |  | Co_bu_IS_R | CAT ACGGTTTGACGTGCTGC |  |
|  |  | Co_bu_IS_P | ATCGGACGTTTATGGGGATGGGTATCC |  |
| *Babesia microti* | *CCTeta* | Bab_mi_CCTeta_F | ACAATGGATTTTCCCCAGCAAAA |  |
|  |  | Ba_mi_CCT_R | GCGACATTTCGGCAACTTATATA |  |
|  |  | Ba_mi_CCT_P | TACTCTGGTGCAATGAGCGTATGGGTA |  |
| *Babesia ovis* | 18SrRNA | Ba_ov_RNA18S_F | TCTGTGATGCCCTTAGATGTC | 92 |
|  |  | Ba_ov_RNA18S_R | GCTGGTTACCCGCGCCTT |  |
|  |  | Ba_ov_RNA18S_P | TCGGAGCGGGGTCAACTCGATGCAT |  |
| *Babesia canis* | 18S rRNA | Ba_ca_RNA18S_F | TGGCCGTTCTTAGTTGGTGG | 104 |
|  |  | Ba_ca_RNA18S_R | AGAAGCAACCGGAAACTCAAATA |  |
|  |  | Ba_ca_RNA18S_P | ACCGGCACTAGTTAGCAGGTTAAGGTC |  |
| *Babesia bovis* | *CCTeta* | Ba_bo_CCTeta_F | GCCAAGTAGTGGTAGACTGTA | 100 |
|  |  | Ba_bo_CCTeta_R | GCTCCGTCATTGGTTATGGTA |  |
|  |  | Ba_bo_CCTeta_P | TAAAGACAACACTGGGTCCGCGTGG |  |
| *Babesia caballi* | *Rap1* | Ba_cab_rap1_ F | GTTGTTCGGCTGGGGCATC | 94 |
|  |  | FBa_cab_rap1_R | CAGGCGACTGACGCTGTGT |  |
|  |  | Ba_cab_rap1_P | TCTGTCCCGATGTCAAGGGGCAGGT |  |
| *Babesia venatorum* (sp. EU1) | 18S rRNA | Bab_EU_RNA18S_F | GCGCGCTACACTGATGCATT | 91 |
|  |  | Bab_EU_RNA18S_R | CAAAAATCAATCCCCGTCACG |  |
|  |  | Bab_EU_RNA18S_P | CATCGAGTTTAATCCTGTCCCGAAAGG |  |
| *Babesia divergens* | *hsp70* | Bab_di_hsp70_F | CTCATTGGTGACGCCGCTA | 83 |
|  |  | Bab_di_hsp70_R | CTCCTCCCGATAAGCCTCTT |  |
|  |  | Bab_di_hsp70_P | AGAACCAGGAGGCCCGTAACCCAGA |  |
| *Theileria* spp. | ARNr 18S | Thei_spp_18S_F | GTCAGTTTTTACGACTCCTTCAG | 213 |
|  |  | Thei_spp_18S_R | CCAAAGAATCAAGAAAGAGCTATC |  |
|  |  | Thei_spp_18S_S | AATCTGTCAATCCTTCCTTTGTCTGGACC |  |
| *Babesia vulpes* | COI | Bab_vulpes_COI_F | ACT TCC AGT GTT AAC AGC AGC | 129 |
|  |  | Bab_vulpes_COI_R | GGA TGT CCG AAG ACC CAG AA |  |
|  |  | Bab_vulpes_COI_P | CTT CTG CAT GTT GCT GGC TGA TAG GCA |  |
| *Hepatozoon* spp. | 18S rRNA | Hepa_spp_18S_F | ATTGGCTTACCGTGGCAGTG | 175 |
|  |  | Hepa_spp_18S_R | AAAGCATTTTAACTGCCTTGTATTG |  |
|  |  | Hepa_spp_18S_S | ACGGTTAACGGGGGATTAGGGTTCGAT |  |
| *Ixodes ricinus* | ITS2 | Ix_ri_ITS2_F | CGAAACTCGATGGAGACCTG | 77 |
|  |  | Ix_ri_ITS2_R | ATCTCCAACGCACCGACGT |  |
|  |  | Ix_ri_ITS2_P | TTGTGGAAATCCCGTCGCACGTTGAAC |  |
| *Dermacentor reticulatus* | ITS2 | De_re_ITS2_F | AACCCTTTTCCGCTCCGTG | 83 |
|  |  | De_re_ITS2_R | TTTTGCTAGAGCTCGACGTAC |  |
|  |  | De_re_ITS2_P | TACGAAGGCAAACAACGCAAACTGCGA |  |
| Tick spp. | 16S rRNA | Tick_spp_16S_F | AAATACTCTAGGGATAACAGCGT | 99 |
|  |  | Tick_spp_16S_R | TCTTCATCAAACAAGTATCCTAATC |  |
|  |  | Tick_spp_16S_P | CAACATCGAGGTCGCAAACCATTTTGTCTA |  |
