# Supplementary material for: High Diversity, Prevalence, and Co-infection Rates of Tick-Borne Pathogens in Ticks and Wildlife Hosts in an Urban Area in Romania
Source: Front Microbiol. 2021 Mar 9;12:645002. doi: 10.3389/fmicb.2021.645002 (PMC7985354; doi:10.3389/fmicb.2021.645002)
Supplement: Supplementary file 4 [file Table_4.docx]

**S4**. Co-infections with tick-borne pathogens in engorged ticks in Cluj-Napoca

| **Location** | **Environment** | **Tick species** | **Percentage of co-infected ticks from total engorged ticks/location/species** | **Life stage**  **(no. of specimens)** | **Pathogen species** |
| --- | --- | --- | --- | --- | --- |
| USAMV Campus | Urban | *I. ricinus* | 3.5 (n=57) | L (n=1)  N (n=1) | *A.ph + Bo.a* |
| USAMV Campus | Urban | *I. ricinus* | 5.3 (n=57) | F (n=1)  N (n=2) | *A.ph + Bo.g* |
| USAMV Campus | Urban | *I. ricinus* | 1.75 (n=57) | L (n=1) | *A.ph + Bo.m* |
| USAMV Campus | Urban | *I. ricinus* | 1.75 (n=57) | N (n=1) | *A.ph + Hepatozoon* spp. |
| USAMV Campus | Urban | *I. ricinus* | 5.3 (n=57) | M (n=1)  L (n=2) | *A.ph + R.h* |
| USAMV Campus | Urban | *I. ricinus* | 3.5 (n=57) | N (n=2) | *A.ph + Theileria* spp. |
| USAMV Campus | Urban | *I. ricinus* | 1.75 (n=57) | N (n=1) | *R.f + Bo.v* |
| USAMV Campus | Urban | *I. ricinus* | 7 (n=57) | F (n=2)  L (n=2) | *A.ph + R.h + Bo.a* |
| USAMV Campus | Urban | *I. ricinus* | 1.75 (n=57) | N (n=1) | *A.ph + R.h + Bo.b/Bo.g* |
| USAMV Campus | Urban | *I. ricinus* | 1.75 (n=57) | N (n=1) | *A.ph + R.h + Bo.m* |
| USAMV Campus | Urban | *I. ricinus* | 1.75 (n=57) | N (n=1) | *A.ph + R.h + Bo.s* |
| USAMV Campus | Urban | *I. ricinus* | 1.75 (n=57) | N (n=1) | *A.ph + R.h + Bo.v* |
| USAMV Campus | Urban | *I. ricinus* | 3.5 (n=57) | M (n=1)  L (n=1) | *A.ph + R.m + Bo.a* |
| USAMV Campus | Urban | *I. hexagonus* | 12.5 (n=8) | F (n=1) | *A.ph + Bo.a* |
| USAMV Campus | Urban | *I. hexagonus* | 12.5 (n=8) | F (n=1) | *A.ph + Bo.g* |
| USAMV Campus | Urban | *I. hexagonus* | 25 (n=8) | F (n=2) | *A.ph + R.m* |
| USAMV Campus | Urban | *I. hexagonus* | 12.5 (n=8) | M (n=1) | *A.ph + R.h + Bo.a* |
| USAMV Campus | Urban | *I. hexagonus* | 12.5 (n=8) | F (n=1) | *A.ph + R.h + Bo.s* |
| Iuliu Hațieganu Park | Urban | *I. ricinus* | 15.2 (n=46) | N (n=5)  L (n=2) | *A.ph + Bo.a* |
| Iuliu Hațieganu Park | Urban | *I. ricinus* | 6.5 (n=46) | N (n=2)  L (n=1) | *A.ph + Bo.g* |
| Iuliu Hațieganu Park | Urban | *I. ricinus* | 2.2 (n=46) | N (n=1) | *A.ph + B.m* |
| Iuliu Hațieganu Park | Urban | *I. ricinus* | 8.7 (n=46) | N (n=1)  L (n=3) | *A.ph + R.h* |
| Iuliu Hațieganu Park | Urban | *I. ricinus* | 2.2 (n=46) | L (n=1) | *R.h + Bo.a* |
| Iuliu Hațieganu Park | Urban | *I. ricinus* | 2.2 (n=46) | L (n=1) | *A.ph + Bo.b/Bo.g* |
| Iuliu Hațieganu Park | Urban | *I. ricinus* | 2.2 (n=46) | N (n=1) | *A.ph + R.h + Bo.a* |
| Iuliu Hațieganu Park | Urban | *I. ricinus* | 2.2 (n=46) | N (n=1) | *A.ph + R.m + Bo.a* |
| Iuliu Hațieganu Park | Urban | *I. ricinus* | 2.2 (n=46) | M (n=1) | *A.ph + R.m + Bo.m* |
| Iuliu Hațieganu Park | Urban | *I. ricinus* | 2.2 (n=46) | M (n=1) | *A.ph + R.h + Bo.b/Bo.g* |
| Iuliu Hațieganu Park | Urban | *I. hexagonus* | 23.1 (n=13) | F (n=3) | *A.ph + Bo.a* |
| Iuliu Hațieganu Park | Urban | *I. hexagonus* | 30.8 (n=13) | F (n=3)  M (n=1) | *A.ph + R.h + Bo.a* |
| Iuliu Hațieganu Park | Urban | *H. punctata* | 9.1 (n=11) | N (n=1) | *A.ph + Bo.a* |
| Iuliu Hațieganu Park | Urban | *H. punctata* | 9.1 (n=11) | L (n=1) | *A.ph + R.m* |
| Alexandru Borza Botanical Garden | Urban | *I. ricinus* | 9.25 (n=54) | N (n=1)  L (n=4) | *A.ph + Bo.a* |
| Alexandru Borza Botanical Garden | Urban | *I. ricinus* | 14.8 (n=54) | M (n=2)  N (n=5)  L (n=2) | *A.ph + R.h* |
| Alexandru Borza Botanical Garden | Urban | *I. ricinus* | 7.4 (n=54) | M (n=1)  L (n=3) | *A.ph + R.m* |
| Alexandru Borza Botanical Garden | Urban | *I. ricinus* | 1.85 (n=54) | N (n=1) | *R.h + N.m* |
| Alexandru Borza Botanical Garden | Urban | *I. ricinus* | 1.85 (n=54) | N (n=1) | *R.h + Theileria* spp. |
| Alexandru Borza Botanical Garden | Urban | *I. ricinus* | 16.6 (n=54) | F (n=3)  M (n=2)  N (n=2)  L (n=2) | *A.ph + R.h + Bo.a* |
| Alexandru Borza Botanical Garden | Urban | *I. ricinus* | 1.85 (n=54) | M (n=1) | *A.ph + R.h + Bo.g* |
| Alexandru Borza Botanical Garden | Urban | *I. ricinus* | 1.85 (n=54) | N (n=1) | *A.ph + R.h + Bo.s* |
| Alexandru Borza Botanical Garden | Urban | *I. ricinus* | 3.7 (n=54) | F (n=1)  N (n=1) | *A.ph + R.h + Bo.v* |
| Alexandru Borza Botanical Garden | Urban | *I. ricinus* | 1.85 (n=54) | L (n=1) | *A.ph + R.m + Bo.a* |
| Alexandru Borza Botanical Garden | Urban | *I. ricinus* | 1.85 (n=54) | F (n=1) | *A.ph + R.h + Theileria* spp. *+ Bo.a* |
| Alexandru Borza Botanical Garden | Urban | *I. ricinus* | 1.85 (n=54) | M (n=1) | *A.ph + R.h + Theileria* spp. *+ Bo.v* |
| Alexandru Borza Botanical Garden | Urban | *I. hexagonus* | 6.6 (n=15) | F (n=1) | *A.ph + Bo.a* |
| Alexandru Borza Botanical Garden | Urban | *I. hexagonus* | 40 (n=15) | F (n=3)  M (n=3) | *A.ph + R.h* |
| Alexandru Borza Botanical Garden | Urban | *I. hexagonus* | 26.6 (n=15) | F (n=3)  M (n=1) | *A.ph + R.h + Bo.a* |
| Alexandru Borza Botanical Garden | Urban | *I. hexagonus* | 6.6 (n=1) | F (n=1) | *A.ph + R.h +Bo.b/Bo.g* |
| Alexandru Borza Botanical Garden | Urban | *I. hexagonus* | 6.6 (n=1) | F (n=1) | *A.ph + R.h + Bo.s* |
| Alexandru Borza Botanical Garden | Urban | *I. hexagonus* | 6.6 (n=1) | M (n=1) | *A.ph + R.h + Borrelia* spp. |

F-females, M-males, N-nymphs, L-larvae; *A. ph - Anaplasma phagocytophilum; Bo.a - Borrelia afzelii; Bo.g - Borrelia garinii; Bo.m - Borrelia miyamotoi; Bo.b - Borrelia bavariensis; Bo.s - Borrelia spielmanii; Bo.v - Borrelia valaisiana; R.h - Rickettsia helvetica; R.m - Rickettsia monacensis; R.f - Rickettsia felis; N.m - Neoehrlichia mikurensis.*
